# Successful Pupation of Small Hive Beetle, *Aethina tumida* (Coleoptera: Nitidulidae), in Greenhouse Substrates

**DOI:** 10.1093/jee/toaa224

**Published:** 2020-09-24

**Authors:** Bram Cornelissen, Peter Neumann, James D Ellis

**Affiliations:** 1 Wageningen Plant Research, Wageningen University and Research, Wageningen, The Netherlands; 2 Institute of Bee Health, Vetsuisse Faculty, University of Bern, Bern, Switzerland; 3 Honey Bee Research and Extension Laboratory, Entomology and Nematology Department, University of Florida, Gainesville, FL

**Keywords:** *Aethina tumida*, honey bee, pupation, greenhouse substrate, invasive species

## Abstract

The small hive beetle, *Aethina tumida* Murray, is an invasive pest that has spread globally. Western honey bees, *Apis mellifera* Linnaeus (Hymenoptera: Apidae), are considered the most important host and infestations can lead to collapse of colonies. Larvae feed on honey, pollen, and brood inside the hive and leave the hive as postfeeding wandering larvae to pupate in the surrounding soil. Other host species include bumble bees, stingless bees, and solitary bees, all of which can facilitate small hive beetle reproduction and are used for greenhouse crop pollination worldwide. Here, we investigated if small hive beetles can complete their life cycle when soil is absent by pupating in plant root-supporting substrates commonly used in greenhouses. Wandering small hive beetle larvae were introduced into containers with coconut fiber, perlite, a mixture of both and stone wool substrates to investigate pupation success and development time. Sand was used as control substrate. In all but one substrate (perlite), small hive beetles developed into adults equally well as they did in the sand. Development time ranged between 23 and 37 d and was not different from that of the control. We showed that small hive beetles can pupate in greenhouse substrates. This could constitute a problem for greenhouse pollination as well as it could facilitate small hive beetle survival in areas which otherwise would be deemed unsuitable or marginal environments for small hive beetles to become established. Our study highlights the opportunistic nature of the small hive beetle as an invasive species.

The small hive beetle, *Aethina tumida* Murray, is an invasive pest of social bee colonies (Ellis and Hepburn 2006, Neumann et al. 2016), which has spread from its native range in Sub-Saharan Africa to all continents except Antarctica (Cornelissen et al. 2019, Schäfer et al. 2019). Small hive beetles reproduce in honey bee nests, usually at cryptic levels that do not damage host colonies (Spiewok and Neumann 2006). Occasionally, reproduction occurs as a mass event, whereby the small hive beetle larvae devour honey bee nest components (bee brood, bee bread, honey, and dead adult bees), often leading to total colony collapse (Ellis 2012). Once reaching the postfeeding stage, small hive beetle larvae migrate out of a hive and burrow into neighboring soil where they pupate in chambers they excavate (Neumann et al. 2016). Small hive beetles can also reproduce in association with nests of bumble bees, stingless bees, and solitary bees (Hoffmann et al. 2008, Neumann et al. 2016, Gonthier et al. 2019).

Honey bees, stingless bees, bumble bees, and solitary bees are all used as pollinators for greenhouse crops. These crops include aubergine, strawberry, courgette, tomato, and bell pepper (Guerra-Sanz 2008), among others. With optimal growing conditions maintained for up to 11 mo a year and the presence of host species for many months, greenhouses could serve as hotspots for small hive beetles, especially in areas where they would otherwise meet environmental constraints limiting their reproduction and survival (Cornelissen et al. 2019). The small hive beetle’s potential use of greenhouses could be limited if the beetle is unable to complete its lifecycle within the structure. Many greenhouse crop systems include substrates other than soil in which plants are grown. Even though small hive beetles can pupate in a variety of soil types, as long as the soils are sufficiently moist (Ellis et al. 2002), their ability to pupate successfully in these greenhouse substrates is currently unknown. Here, we investigated if substrates commonly used in greenhouses are suitable pupation media for small hive beetles. We hypothesized that small hive beetle larvae would be able to pupate in these substrates, making greenhouses potentially suitable sites for small hive beetle reproduction and survival.

## Materials and Methods

Adult small hive beetles (*n* = 44) were manually collected 11 August 2016 from naturally infested local honey bee colonies managed according to standard practices for the region at the University of Florida, Honey Bee Research and Extension Laboratory, Bee Biology Unit (Gainesville, FL, 29°37′37.1″N 82°21′22.6″W). The adult small hive beetles were divided into two groups and placed into separate cubic plastic boxes (approximately 2 liter per box), each containing 400 g of standard small hive beetle food mixture (Neumann et al. 2013). The adults were left to oviposit and the boxes were maintained at 25°C and constant darkness during the experiment. The boxes were checked twice weekly, at which time moist tissue paper and additional food were added ad libitum. The tissue paper was moistened to near saturation with tap water and was used to raise the humidity in the breeding boxes to facilitate larval hatching (Neumann et al. 2013). On 1 September 2016, ample wandering larvae (Neumann et al. 2013) were available to start the pupation experiment.

Three greenhouse substrates were selected for this investigation (purchased in dry form, at Gator Hydroponics, Gainesville, FL): one organic (coir or coconut fiber—Cocogro) and two inorganic substrates (stone wool and perlite), all of which are commonly used in soilless plant cultures (Bar-Tal et al. 2019). Furthermore, we used a mixture of coconut fiber and perlite in a 70/30 ratio by mass, as it is also used in greenhouse cultures (Bar-Tal et al. 2019). We used sand (Quickrete premium playsand) as a positive control because small hive beetles readily pupate in sand (Ellis et al. 2002). The substrates were put into transparent plastic pupation containers (1.6 liter), with a minimum depth of 10 cm of substrate available for small hive beetle pupation. A total volume of 1,100–1,200 ml of substrate was used. The moisture levels of the different substrates varied due to the differences in the water holding capacity of the substrates (Table 1). Principally, the substrates were saturated with tap water, after which excess water was left to soak for 10 min and then drain for another 10 min.

**Table 1.** Substrate composition per container used for the in vitro pupation of small hive beetles, *Aethina tumida*

Substrate	Weight of substrate (g)	Water (ml)
Perlite	100	236
Coconut fiber/perlite	70/30	411
Coconut fiber	100	600
Stone wool	90	766
Sand	1,250	100

Substrate weight and water volume summed to a total volume of 1,100 to 1,200 ml per container.

Twenty-five small hive beetle wandering larvae were added to each pupation container, totaling 100 larvae per substrate distributed over four replicate containers per each of the five treatments. Thereafter, the containers were placed in an incubator at 25°C and total darkness for the duration of pupation. The containers were checked daily, for 37 d, for dead larvae and emerged adults until 9 October 2016, on which day all containers were checked for remaining live adult small hive beetles by filtering the soil.

A generalized linear model (GLM) with a betabinomial distribution was used to compare emergence rates aggregated for containers with substrate as the fixed factor. We then performed pairwise comparisons between emergence rates of the substrates tested. Small hive beetle larvae that drowned within the first 5 d after exposure to the substrates were omitted from the analysis. All hypothesis tests were likelihood ratio tests (LRT). Similarly, development time (day adult emerged *minus* day larvae added to substrate) was compared between the different substrates with a univariate GLM with containers as a random factor. Pairwise comparisons between substrates were performed and estimated means generated from the models.

## Results

Within 5 d after introduction to the substrates, 44 larvae (range: 5–13) drowned in the excess water accumulated at the bottom of the perlite containers. Similarly, four larvae had drowned in one of the containers of stone wool. The GLM analysis for the emergence of small hive beetles showed significant differences among substrates (df = 4, LRT = 27.267, *P* < 0.001). The pairwise comparison showed that emergence rates of small hive beetles were similar for all substrates, except for the perlite substrate (Table 2). The emergence rates in stone wool, coconut fiber, and the mixture were not different from that in the control substrate (sand, *P* > 0.05). Typical small hive beetle pupation chambers could be observed through the container wall in all substrates, except perlite. Here, the remaining larvae did not pupate and eventually died. Significant differences were observed in the development time of small hive beetles pupating in the various substrates (*F* = 6.355, df = 3, *P* < 0.001, see Table 2). Development time in coconut fiber and stone wool substrates were significantly shorter than in the control substrate (*P* < 0.05), but similar to the coconut fiber/perlite mixture. The latter was not different from the control substrate (*P* > 0.05). The first adult small hive beetles emerged from the coconut fiber substrate after 23 d. The last adults to emerge were observed in the sand, 37 d after the larvae were exposed to the substrate.

**Table 2.** Estimated mean emergence rates (%) and development time (days) of small hive beetles, *Aethina tumida*, pupae in the tested greenhouse substrates and in sand as a positive control (emergence rate: GLM, df = 4, LRT = 27.267, *P* < 0.001, development time: GLM, *F* = 6.355, df = 3, *P* < 0.001)

Substrate	Emergence rate (± SE) (%)	Development time (± SE) (d)
Perlite	0 (0.0)^a^	Not applicable
Coconut fiber/perlite	68.1 (12.0)^b^	28.5 (0.42)^ab^
Coconut fiber	79.2 (10.4)^b^	27.7 (0.43)^b^
Stone wool	87.6 (8.1)^b^	27.8 (0.33)^b^
Sand	81.5 (8.6)^b^	29.5 (0.31)^a^

*n* = 4 replicate containers and *n* = 456 and *n* = 317 larvae for emergence rate and development time, respectfully. Larvae that drowned in the first 5 d were omitted from the analysis. Column means with the same letter are not different at *P* ≤ 0.05.

## Discussion

Our data clearly show that small hive beetles can pupate in substrates used in greenhouse settings. In fact, small hive beetles pupated equally well in coconut fiber, coconut fiber + perlite, stone wool, and sand (the control substrate) with the emergence rates varying between 68.1 and 87.6%. The development time for rockwool and coconut fiber was shorter than the control substrate. Several studies obtained comparable results in various substrate types at similar temperatures (Cornelissen et al. 2019; Supp Table 1 [online only]), thereby indicating that all of the tested substrates, except perlite, are suitable for completion of the small hive beetle life cycle. In comparison with the other substrates, perlite (an amorphous volcanic glass) has an extremely low density and does not form a consistent mass. The perlite substrates were composed of separate lumps of up to 1 cm in size, which probably could not support certain physical aspects required by small hive beetles for pupation. For instance, wandering larvae were unable to make pupation chambers (Neumann and Elzen 2004) and would fall to the bottom, unable to crawl back into the substrate. Furthermore, several days into the experiment, water would accumulate at the bottom of the containers, in which several larvae drowned. Drowning of larvae (*n* = 4) also occurred in one container with stone wool. The cause of this was not related to the suitability of the substrate, but rather because the stone wool did not touch the bottom of the container entirely.

Our data further suggest that small hive beetles could cause problems to bees used to pollinate horticultural crops grown in greenhouses. However, no such large-scale problems have been reported in bumble bee pollinated greenhouse crops in the United States because the small hive beetle became established in 1996. At the same time, no information exists on the potential risks for other bee species, such as *Bombus terrestris* Linnaeus (Hymenoptera: Apidae), used for crop pollination outside of the United States. Furthermore, the risk for honey bee pollination units could be higher, as small hive beetles could already be residing in a colony before they enter a greenhouse. Although the use of honey bees for greenhouse pollination in the United States might be minimal, they are used at a larger scale in other countries. For instance, approximately 5,000 honey bee colonies are used annually for greenhouse and seed pollination in the Netherlands (Blacquière et al. 2009). Moreover, social bee colonies could be prone to small hive beetle infestation in greenhouses given that foraging and environmental conditions in greenhouses are generally suboptimal for pollinator units (Guerra-Sanz 2008).

In a broader context, our data suggest an increased ability and likelihood for small hive beetles to complete their life cycles in greenhouses, thereby constituting potential hot spots for this beetle in regions, which would otherwise be marginal or unsuitable for establishment of this invasive species (Cornelissen et al. 2019). In conclusion, we demonstrated that the small hive beetle can pupate in a variety of substrates, which is consistent with the opportunistic nature of this invasive species (Gonthier et al. 2019).
